# *Mycobacterium tuberculosis *spoligotypes and drug susceptibility pattern of isolates from tuberculosis patients in South-Western Uganda

**DOI:** 10.1186/1471-2334-11-81

**Published:** 2011-03-31

**Authors:** Joel Bazira, Benon B Asiimwe, Moses L Joloba, Freddie Bwanga, Mecky I Matee

**Affiliations:** 1 Department of Microbiology, Faculty of Medicine, Mbarara University of Science and Technology, Mbarara, Uganda; 2 Department of Medical Microbiology, School of Biomedical Sciences, College of Health Sciences, Makerere University, Kampala, Uganda; 3 Department of Microbiology and Immunology, School of Medicine, Muhimbili University of Health and Allied Sciences, Dar es Salaam, Tanzania

## Abstract

**Background:**

Determination of the prevalence and drug susceptibility of the *M. tuberculosis *strains is important in tuberculosis control. We determined the genetic diversity and susceptibility profiles of mycobacteria isolated from tuberculosis patients in Mbarara, South Western Uganda.

**Methods:**

We enrolled, consecutively; all newly diagnosed and previously treated smear-positive TB patients aged ≥ 18 years. The isolates were characterized using regions of difference (RD) analysis and spoligotyping. Drug resistance against rifampicin and isoniazid were tested using the Genotype^® ^MDRTBplus assay and the indirect proportion method on Lowenstein-Jensen media. HIV-1 testing was performed using two rapid HIV tests.

**Results:**

A total of 125 isolates from 167 TB suspects (60% males) with a mean age 33.7 years and HIV prevalence of 67.9% (55/81) were analyzed. Majority (92.8%) were new cases while only 7.2% were retreatment cases. All the 125 isolates were identified as *M. tuberculosis *strict sense with the majority (92.8%) of the isolates being modern strains while seven (7.2%) isolates were ancestral strains. Spoligotyping revealed 79 spoligotype patterns, with an overall diversity of 63.2%. Sixty two (49.6%) of the isolates formed 16 clusters consisting of 2-15 isolates each. A majority (59.2%) of the isolates belong to the Uganda genotype group of strains. The major shared spoligotypes in our sample were SIT 135 (T2-Uganda) with 15 isolates and SIT 128 (T2) with 3 isolates. Sixty nine (87%) of the 79 patterns had not yet been defined in the SpolDB4.0.database. Resistance mutations to either RIF or INH were detected in 6.4% of the isolates. Multidrug resistance, INH and RIF resistance was 1.6%, 3.2% and 4.8%, respectively. The *rpoβ *gene mutations seen in the sample were D516V, S531L, H526Y H526D and D516V, while one strain had a Δ1 mutation in the wild type probes. There were three strains with *katG *(codon 315) gene mutations only while one strain showed the *inhA *promoter gene mutation.

**Conclusion:**

The present study shows that the TB epidemic in Mbarara is caused by modern *M. tuberculosis *strains mainly belonging to the Uganda genotype and anti-TB drug resistance rate in the region is low.

## Background

Uganda ranks 16th among the world's 22 countries with the highest tuberculosis burden in the world [1]. The country had more than 132,000 TB cases in 2007, with an estimated incidence rate of 330 per 100,000 people with greater Mbarara contributing about 26% of all TB cases. This region has been heavily affected by the TB/HIV epidemic. In 2005, the case notification for Mbarara was 175/100,000 people compared to the 147/100,000 people for Uganda [2]. In 2008 the TB/HIV co-infection rate for Mbarara was 65% [3]

To date, there are very limited data available pertaining strains circulating in Mbarara yet evidence indicates that *M. tuberculosis*' ability to spread varies from strain to strain and those different strains have different geographical and/or host specificities [4,5]. The presence of Human immunodeficiency virus (HIV) has caused an increase in *Mycobacterium tuberculosis *complex (MTC) infection [6] and rapid progression of the infection [7] and is also known to increase MTC transmission rates at the community level, further threatening the health and survival of HIV sero-negative individuals as well [8].

Since the discovery of DNA polymorphisms in *M. tuberculosis*, molecular typing of strains has become an invaluable tool for the study of epidemiology of TB. Some of the applications include; predicting transmission rates and identifying dominant strains associated with outbreak [9] severe disease [10] and drug resistance.

Comparative-genomics approaches greatly enhanced our understanding of the mechanisms of insertion and deletion of DNA and the resulting distribution of variable regions around the genomes of tubercle bacilli [11-13]. There are 20 variable regions of which 14 regions of difference (RD1 to RD14) were found to be absent from Bacillus Calmette-Guérin (BCG) Pasteur relative to *M. tuberculosis *H37Rv [13-15]. Six regions, H37Rv-related deletions (RvD1 to RvD5 and *M. tuberculosis *specific deletion 1 (TbD1) are absent from the *M. tuberculosis *H37Rv genome relative to other members of the *M. tuberculosis *complex. Based on the presence or absence of the TbD1 region, *M. tuberculosis *strains can be divided into "ancestral" and "modern" types. The Beijing, Haarlem, and African strains responsible for major epidemics are modern types [11,16].

Clustered regularly interspaced short palindromic repeats (CRISPRs) are repetitive structures in bacteria and archaea composed of exact repeat sequences 24 to 48 bases long separated by unique spacers of similar length [17,18]. The CRISPR sequences appear to be among the most rapidly evolving elements in the genome, to the point that closely related species and strains, sometimes more than 99% identical at the DNA level, differ in their CRISPR composition [19,20]. Direct Repeat loci (DR) are members of the CRISPR [21]. The Direct Repeat locus consists of alternating identical DRs and variable spacers can be assessed using the spoligotyping fingerprinting methodology. Variability in the direct repeat locus of *M. tuberculosis *[22] most likely occurs by one of three mechanisms--homologous recombination between neighboring or distant direct variable repeats, IS-mediated transposition, and DNA replication slippage [22].

Drug resistance among mycobacteria is a threat to the treatment of tuberculosis globally and threatens to reverse the gains made so far in the fight against tuberculosis. Several competing technologies have been proposed for rapid detection of drug resistant tuberculosis. Some commercial assays are currently available including INNO-LiPA Rif.TB (Innogenetics N.V, Ghent, Belgium) and GenoType^® ^MTBDR (HAIN Lifesciences GmbH, Nehren, Germany) [23]. The new version of the latter assay (GenoType^® ^MTBDRplus), targeting the *rpoB *gene associated with the resistance to rifampicin (RIF) and both genes (*katG *and *inhA*) commonly associated with the resistance to isoniazid (INH) has been evaluated mainly on cultures and clinical specimens in various low incidence settings, demonstrating excellent specificity and good concordance with phenotypic drug susceptibility test (DST) results [24,25].

The current study was carried out in the Mbarara region of South-Western Uganda between May 2007 and April 2008 in order to know the clones of *M. tuberculosis *and their drug resistance patterns.

## Methods

### Study setting

**Figure 1:**
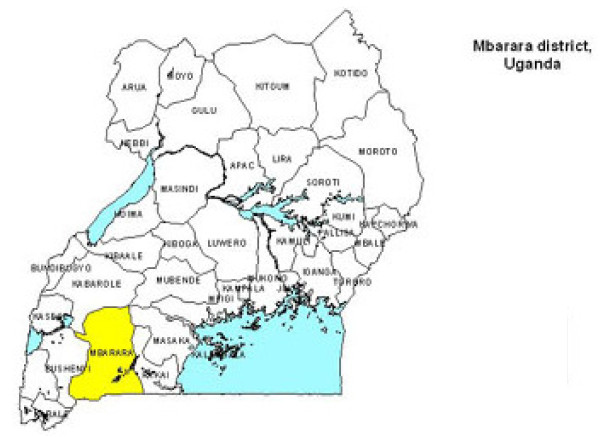
**Map of Uganda showing former greater Mbarara district, the study area**.

The study was conducted between May 2007 and April 2008 in the former greater Mbarara in the South- western region of Uganda. Greater Mbarara is a rural region that is currently made up of four districts namely Ibanda, Isingiro, Kiruhura and Mbarara (Figure 1).

### Study design

This was a cross sectional study in which we enrolled, consecutively, all consenting smear-positive newly diagnosed and retreatment TB patients aged ≥ 18 years. Three consecutive sputum samples (spot, early morning and spot) were taken from each patient according the Uganda National TB and Leprosy guidelines. Only the sample with the highest ZN smear grade for each patient was further processed and cultured. Samples were stored at 4°C at the recruitment clinics, in any case for not more than 48 hours, until transported in a cold box to the National tuberculosis reference laboratory (NTRL) in Kampala for processing and culture.

### Sputum sample processing

Specimens (2.5-10 ml) were processed by the standard N-acetyl L-cystein (NALC)-NaOH method [26] and concentrated at 4000 × *g *for 15 minutes. The sediment was reconstituted to 2.5 ml with phosphate buffer pH 6.8, to make the inoculum for the smears and cultures.

### Culture and identification

Two Löwenstein-Jensenslants, one containing 0.75% glycerol and the other containing 0.6% pyruvate were inoculated with the sediment and incubated at 37°C and examined weekly for growth. Cultures were considered negative when no colonies were seen after 8 weeks incubation.

### DNA Extraction

Isolates were harvested and DNA extracted using a standardized protocol [27].

### RD analysis

RD analysis for 16SrRNAS, RD4, RD 9, RD12 and TbD1 was performed for speciation of the isolates was done at the department of Medical Microbiology, Makerere University College of Health Sciences as previously described [28].

### Spoligotyping

Standard spoligotyping was performed as previously described [29] using a commercially available kit (Isogen Bioscience BV, Maarssen, The Netherlands).

### Conventional drug susceptibility testing

The indirect proportion method on Lowenstein-Jensen media was performed by the NTLP for patient management at the following final drug concentrations: rifampicin, 40 μg/ml and isoniazid, 0.2 μg/ml. The NTLP kindly provided the results for comparison with the kit results.

### GenoType^® ^MTBDRplus assays

Identification of mutations in *rpoB*, *katG*, and *inhA *genes associated with resistance to RIF and INH was performed on the mycobacterial cultures according to the manufacturer's recommendations. Briefly, heat thermolysates of cultures were obtained by heating cultures suspended in Tris-EDTA (TE) at 80°C for two hours followed by incubation in an ultrasonic bath for 5 minutes. PCR and subsequent hybridization steps were performed according to manufacturer's recommendations. Thereafter, strips were attached to the evaluation sheet, read and interpreted. For quality control we included known fully sensitive and resistance isolates in each run.

### HIV testing

HIV-1 testing was performed using two rapid HIV tests, Unigold Recombinant HIV (Trinity Biotech, Wicklow, Ireland) and Determine HIV-1/2 (Abbott, Tokyo, Japan). Samples were tested first with Abbot Determine. Positive samples were confirmed with Unigold, while discordant results were resolved by a third rapid test kit, HIV-1/2 Stat-Pak (ChemBio, Medford, NY). Pre and post test HIV counseling was done for all consenting individuals.

### Data analysis

Socio-demographic data was entered into the computer using Microsoft Excel 2000 software and then exported to SPSS version 10 for analysis. All spoligotyping data were digitized and analyzed with the BioNumerics software, version 5.0 (Applied Maths, Kortrijk, Belgium). Spoligotype identification was carried out according to SpolDB4 [30] and by the freely accessible MIRU-VNTR*plus *web database [31]. The latter comprises spoligotyping data of a reference strain collection of validly described Mycobacterium tuberculosis complex (MTBC) genotypes. Labels for major phylogenetic families were assigned according to signatures provided in SpolDB4. Univariate analysis of categorical variables utilized percentages while means and standard deviations were calculated for continuous variables. The median and range was presented whenever continuous data was skewed. Chi squares were computed in STATA version 11 and a P value of < 0.05 was considered evidence of significant difference.

### Ethical considerations

This study received ethical clearance from the faculty research and ethics committee of the faculty of medicine of Mbarara University of Science and Technology, the institutional review board of Mbarara University of Science and Technology and the Uganda National Council for Science and Technology. Patients were identified and managed according to Uganda NTLP guidelines [32]. Informed consent to participate in the study as well as permission to use isolates from samples provided were obtained from all enrolled participants.

## Results

### Study population and samples

We enrolled a total of 167 sputum smear positive tuberculosis suspects presenting at the various TB clinics in the greater Mbarara during the study period. The samples were graded depending on AFB count in the specimen according to the WHO recommendations [33]. Of the 167 samples that were cultured 140 (84%) grew, 14 (8%) had no growth and 13 (8%) were contaminated. Of the 140 cultures 15 (10.7%) did not have full demographic data and were therefore not available for the study leaving 125 isolates. Of the 125 patients with viable isolates, majority 116 (92.8%) were newly diagnosed and 9 (7.2%) had previous history of TB treatment. Of these patients, 50 were females and 75 were males with a mean age of 33.7 years.

### RD analysis

All the 125 isolates that were available for RD analysis were identified as *M. tuberculosis *sensu stricto. Seven isolates were found to have all the RD loci conserved including TbD1 and were therefore ancestral strains (Table 1).

**Table 1:** Species identification and PCR results of the *M. tuberculosis *complex isolates

*M. tuberculosis *Complex	Targeted PCR Locus				
	
	16rDNA	RD4	RD9	RD12	TbD1
Ancestral M. tuberculosis*(n = 07)	+	+	+	+	+

Modern *M. tuberculosis*^† ^(n = 118)	+	+	+	+	-

*Defined as possessing the TbD1 locus intact; †Defined as possessing the TbD1 deletion;

+ = locus intact, - = locus deleted

### Spoligotyping

**Figure 2:**
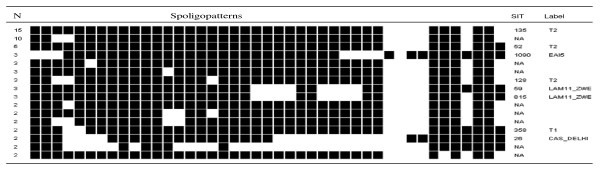
**Spoligotype pattern of clustered *M. tuberculosis *strains in the study**. SIT, spoligotype international type; N, number of isolates, filled boxes represent positive hybridization while empty boxes represent absence of spacers; label defines the lineage/sub lineage; NA, not available in SpolDB4.0.

All the isolates had spoligotypes characteristic of *M. tuberculosis*. The isolates gave 79 different spoligopatterns, with an overall diversity of 63.2%. A total of 62 (49.6%) isolates, were grouped into 16 clusters consisting of 2-15 isolates each, while the remaining 63 (51.4%) isolates did not cluster. Among the 16 clusters, three included five or more isolates each and were defined as major spoligotypes in this study, while minor spoligotypes, on the other hand, were defined as spoligotype international types (SITs) that contained less than five isolates per cluster (Figure 2). Spoligotypes that neither clustered nor matched any existing pattern in the SpolDB4 database were defined as orphans. Of the 79 patterns observed in this study, 69 were true orphans.

The family assignment showed that 59.2% of the isolates belonged to the Uganda genotype, 7.2% to the CAS_DELHI, 6.4% to the Latin American Mediterranean (LAM), 5.6% to the East-African Indian (EAI) family, 4% belonged to the Cameroon family, 2.4% to the Ghana family and 13.6% were not assigned to any family.

Comparison of the spoligotypes in our study with SpolDB4.0 allowed differentiation between ubiquitous types (SIT 26-CAS_DELHI, SIT 52-T2, SIT 53- T1, SIT 54-MANU2, SIT 59- LAM11_ZWE, SIT356- CAS, SIT 358-T1, SIT 420-T2, SIT 815- LAM11_ZWE, SIT 1090 - EAI5 and SIT 1572-T2) and those believed to be endemic in Uganda (SIT 128- T2 and SIT 135 -T2).

A majority (59.2%) of the isolates lacked hybridization to either spacer 40 alone (Uganda genotype II) or to both 40 and 43 (Uganda genotype I). These Uganda genotype strains formed 8 clusters (ranging 2 to 15 isolates). Nine strains were Uganda genotype I while seventy seven were Uganda genotype II. Forty eight Uganda genotype strains did not cluster.

### HIV sero-status

Fifty five patients were HIV sero-positive, twenty six were HIV sero negative and forty four had unknown sero-status because they did not consent to HIV testing. There was no significant difference (P = 0.116) in sero-status between female and male patients in the study.

An analysis of the predominant spoligotypes in HIV sero-positive and sero-negative patients showed that 28/55 (50.9%) of sero-positive individuals carried strains of the Uganda family, while only 12.7% carried the LAM strains,3.2% Delhi/CAS strains, and 5.6% carried unique strains. Furthermore, 19/26 (73.1%) of the HIV sero-negative individuals carried strains of the Uganda family, while only 3.8% carried the LAM strains,3.8% Delhi/CAS strains, and 15.4% carried unique strains. Of those who did not consent for HIV testing 27/44 (61.4%) carried the Uganda family of strains while only 2.3% carried the LAM strains,11.4% Delhi/CAS strains, and 13.6% carried unique strains.

### Drug susceptibility patterns

Susceptibility testing showed that six isolates were resistant to rifampicin, and four to isoniazid, with two isoniazid resistant isolates being rifampicin resistant as well, hence MDR. All the two MDR strains were isolated from patients below 39 years of age, all being HIV sero-positive females. Surprisingly, the MDR strains were isolated from newly presenting patients. Four of the 74 isolates of Uganda family were resistant to at least one drug while two of the Delhi/CAS family was resistant to at least one drug. There were two MDR isolates, one belonging to the Delhi/CAS family and the other to Uganda family. The rifampicin resistant isolates displayed three types of mutations: three isolates had a mutation at position D516V, two had S531L, while one of the isolates with D516V had a further two mutations, H526Y and H526D in the *rpoβ *gene. Only one strain had a Δ1 mutaion in the wild type probes and, according to the kit manufacturer's recommendation, was considered resistant. There was no significant association between drug resistance and any lineage. A summary of drug susceptibility pattern of the isolates with the spoligotypes is shown in Table 2.

**Table 2:** Association of drug susceptibility pattern of the isolates with Spoligotypes

Susceptibility Profiles	Lineages							
		
	Uganda	E AI	Dehli/CAS	LAM	Ghana	Cameroon	Others	*P *Value
RIF Resistant	3	0	1	1	0	0	1	0.894

RIF Sensitive	71	7	9	8	3	5	16	

INH resistant	1	0	2	1	0	0	0	0.46

INH sensitive	73	7	8	8	3	5	17	

Sensitive to both drugs	70	7	8	8	3	5	16	0.076

Any Resistance	4	0	2	1	0	0	1	

MDR	1	0	0	1	0	0	0	0.436

Non MDR	73	7	10	8	3	5	17	

## Discussion

Deletion analysis using 16SrRNAS, RD4, RD 9, RD12 and TbD1 revealed that all the strains investigated were *M. tuberculosis*. This finding is comparable to that of the study by Byarugaba et al 2009 [34] which found no *M. bovis *among the 75 MTC isolates from the mainly cattle rearing communities of greater Mbarara. This could be a result of improved animal husbandry in Mbarara, or due to improved practices of the people such as boiling of milk before consumption. On the other hand, it may be due to the fact all of our samples were pulmonary sputum, which might have limited the chances of culturing *M. bovis*.

The diversity of the *M. tuberculosis *found in the present study (63.2%) is high compared to 16.6% described from a study in central Uganda [35], which may indicate geographical differences in the epidemiology of TB in Uganda. Another hypothesis could be because of differences in the population density. Central Uganda has the major cities of the country and therefore has a high transmission rate, hence limited diversity. Mbarara is a big district, sparsely populated because of the main economic activity: cattle keeping. In such an area, it is possible to have less transmission and a highly diverse bacterial population. This is not easily determined using spoligotyping alone and would therefore require more discriminatory techniques such as MIRU and restriction fragment length polymorphism (RFLP) to confirm this.

In this study 69 (87%) of the spoligopatterns could not be typed on the basis of the existing SpolBD4 database. This indicates the current absence of knowledge on the genetic diversity of *M. tuberculosis *strains in from this region. This therefore calls for more clinical epidemiological studies in different regions of not only Uganda but Africa at large so as to clearly understand the genetic diversity of the TB epidemic on the continent.

Majority (59.2%) of the strains in our sample were of the Uganda genotype, a finding which is in keeping with those of earlier studies in Uganda [36-38] as opposed to findings from the surrounding East African countries[39,40]. For example a study in Kenya [39] found only eight (11%) of 73 isolates to be of the Uganda family while in northern Tanzania [40] only four (3%) of 130 strains were T2-Uganda. Collectively these findings are in agreement with those of other studies showing a tendency for local genotypes to form a greater proportion of the circulating strains in different parts of Africa [41-44]. These results indicate that each of the major lineages of *M. tuberculosis *have evolved to most efficiently transmit within an original human population. These results are further in agreement with other findings where it was noted that different strains of *M. tuberculosis *have adapted to specific human populations, and that such local strains are more likely to transmit compared to others [4,45].

The relative frequencies of major *M. tuberculosis *spoligotype families were in range with the overall frequencies in Uganda and other East African countries [35,36,39,40,46]. The largely predominant Uganda family identified in more than 59% of the strains in our study is ubiquitous in this country. Both its high degree of dissemination and its preponderance among the new (shared as well as orphan) patterns are manifestations of the current adaptive evolution of the Uganda genotype in this setting. Other significant spoligotypes in our study were LAM (7.2%), CAS (8%), EAI (5.6%), Cameroon (4%) and Ghana (2.4%). A study by Asiimwe et al 2008 from Kampala showed proportions of CAS1-Kili (3.5%), LAM9 (2.6%), CAS1-Delhi (2.6%), LAM3/S (1.7%), CAS1 (1.7%), and LAM11-ZWE (1.5%). In comparison to other studies in the region, the CAS, LAM and EAI families were reported at 37%, 22% and 17% respectively of a total of 147 isolates in a study in Dar es Salaam, Tanzania [46]; while in northern Tanzania, the most predominant families were CAS-Kili (30%), LAM11-ZWE (14.6%), EAI (6.2%), Beijing (5.4%), and CAS1-Delhi, T1 and LAM9 at 3.8% [40]. In Kenya, on the other hand, 35.6% of 73 isolates were of the CAS family, while 11% were LAM [39]. These studies show more success of the CAS, LAM and EAI families in the neighboring East African countries, while in Central and Western Uganda, the Uganda family of strains predominates.

We found resistance to isoniazid and rifampicin to be 3.2% and 5.6% respectively, while MDR was 1.6% (2/125). Our result show differences compared with findings that were obtained in the last National anti-tuberculosis drug resistance survey in Uganda of 1996-97 that indicated a primary resistance to isoniazid of 6.7%, that to rifampicin at 0.8%, and MDR of 0.5% [47]. More recently, a study in peri-urban Kampala showed resistance to isoniazid of 8.1%, rifampicin resistance of 4.4% and MDR was found to be 4.4% [35]. These differences may probably be due to sampling strategy employed in each study and the numbers involved. While the National survey randomly sampled districts in Uganda, the peri-urban study in Kampala looked at a single division known to be the second most TB burdened in the city, while the current study sampled patients from various villages of a rural district in western Uganda.

Studies from neighbouring East African countries show varied results. In a study in Rwanda, resistance to isoniazid was found at 6.2%, that to rifampicin was 3.9% with all rifampicin resistant isolates being multidrug-resistant [48]. In northern Tanzania, on the other hand, a study of 111 isolates showed that 9.9% were resistant to isoniazid, 2.7% to rifampicin, while MDR was 2.7%[40]. Generally, the drug resistance rates in the current study are fairly within the range of those found in previous studies both in-country and around the region. However there is evidence of an increase in the MDR rate in Uganda in the last two studies compared to the first National survey albeit on smaller samples. Although a number of patients were not tested for HIV and could be dually infected, two thirds of those tested for were infected by both HIV and TB, a common trend in sub Saharan Africa [49].

Despite the high prevalence of the Uganda family there was no significant association with anti-TB drug resistance (*P*-value = 0.076) This finding in agreement with the finding by Asiimwe et al [35] who found that there was no significant difference in the resistance pattern of the predominant T 2 Uganda genotype versus the Non T family. This therefore implies that T2 Uganda might not be the driving force of anti-tuberculosis drug resistance in this community Mutations in codon 315 of the *katG *gene were the only ones found in our study. These mutations are found in the vast majority of isoniazid-resistant isolates [50,51]. However, the frequency of codon 315 mutations in isoniazid-resistant isolates in other populations has been reported to range from 35% to 97% [52]. Mutations in codon 315 do not significantly decrease the peroxidase activity of the *katG *gene product, but do decrease its ability to activate isoniazid[53]. These features allow the mutants to maintain the peroxidase activity required for virulence, and to resist killing by isoniazid. Such isolates often display resistance to only lower levels of isoniazid, and resistance to higher levels appears to correlate with loss of catalase activity or acquisition of mutations in multiple genes implicated in isoniazid resistance, e.g., *inhA *or *ahpC*[54,55].

The most frequent mutation in the *rpoB *codons in our study was 516 (43%). As opposed to mutation 531 which is the most commonly observed mutation in rifampicin-resistant isolates in many parts of the world, e.g., in Brazil (54%) [56], the USA (35%) [57], India (38.7%) [58], Germany (65%) [59] and Australia (52%) [60].

Differences in the frequencies of mutations in the 516, 526 and 531 codons of the *rpoB *gene among isolates of the Beijing, Haarlem and LAM families may primarily reflect differences in mutational frequencies or the relative fitness of the mutations in the strain families, as opposed to a possible sampling bias caused by extensive transmission of individual MDR strains.

Overall, the data indicate that the frequencies of individual mutations in the genes associated with rifampicin and isoniazid resistance vary among isolates belonging to different genotype families. Such variation may influence the performance of molecular diagnostic tests designed to detect mutations associated with drug resistance in *M. tuberculosis *isolates. This emphasises the importance of validating the performance of a diagnostic test in the population being tested. The biological significance of the predominance of certain mutations in particular genotype families remains to be determined.

## Conclusions

This study provides an insight into the *M. tuberculosis *strains circulating in the rural district of Mbarara, South Western Uganda. We have shown that the majority of the Mycobacteria in Mbarara are modern *M. tuberculosis *with a wide diversity of spoligotypes and a predominance of the Uganda genotype. Additionally, strain types in our sample were not associated with drug resistance.

## Competing interests

The authors declare that they have no competing interests.

## Authors' contributions

JB participated in the planning of the study, acquisition of samples and demographic data, culture and isolation of mycobacteria, molecular assays and drafting of manuscript; BBA participated in molecular assays, data analysis and drafting of the manuscript, MLJ participated in general supervision of the study and critical revision of the manuscript, FB participated in performing the HAIN assays, MM participated in the conception of the study, general supervision of the study, critical revision of manuscript. All authors read and approved the final manuscript.

## Pre-publication history

The pre-publication history for this paper can be accessed here:

http://www.biomedcentral.com/1471-2334/11/81/prepub
